# The Integrative Role of Berberine in Gut Microbiota Modulation and Cardiometabolic Outcomes: A Systematic Review of Randomised Clinical Trials

**DOI:** 10.3390/nu18121858

**Published:** 2026-06-09

**Authors:** Adelin-Rareș Candrea, Laura Ioana Gavrilaș, Oleg Frumuzachi, Andrei Mocan, Mihai Babotă, Gianina Crișan

**Affiliations:** 1 Department of Pharmaceutical Botany, Faculty of Pharmacy, “Iuliu Hațieganu” University of Medicine and Pharmacy, 23 Gheorghe Marinescu Street, 400337 Cluj-Napoca, Romania; adelin.rare.candrea@elearn.umfcluj.ro (A.-R.C.); oleg.frumuzachi@elearn.umcluj.ro (O.F.); andrei.mocan@umfcluj.ro (A.M.); gcrisan@umfcluj.ro (G.C.); 2 2nd Department, Faculty of Nursing and Health Sciences, “Iuliu Hatieganu” University of Medicine and Pharmacy, 23 Gheorghe Marinescu Street, 400337 Cluj-Napoca, Romania; 3 Department of Development, Innovation and Research, Romanian Dietitians Association, 17A, Janos Zsigmond Street, 400347 Cluj-Napoca, Romania; 4 Research Centre of Medicinal and Aromatic Plants, “George Emil Palade” University of Medicine, Pharmacy, Science and Technology of Târgu Mureș, 38 Gheorghe Marinescu Street, 540139 Targu Mures, Romania; mihai.babota@umfst.ro; 5 Department of Pharmaceutical Botany, Faculty of Pharmacy, “George Emil Palade” University of Medicine, Pharmacy, Science and Technology of Târgu Mureș, 38 Gheorghe Marinescu Street, 540139 Targu Mures, Romania

**Keywords:** gut microbiota, berberine, randomised controlled trial, dysbiosis, microbiota modulation

## Abstract

**Background/Aim**: Berberine, an isoquinoline alkaloid widely used in traditional medicine, has attracted considerable interest for its capacity to modulate the gut microbiota and improve cardiometabolic outcomes. Although preclinical evidence is promising, no systematic review has previously synthesised evidence from randomised controlled trials (RCTs) in humans. This review aimed to evaluate the effects of berberine supplementation on gut microbiota and to explore associated cardiometabolic, inflammatory, and immunological changes. **Methods**: Prospectively registered in PROSPERO (CRD42024524143) and conducted in accordance with PRISMA guidelines, this review searched PubMed, Web of Science, Scopus, and Embase from inception to 10 January 2026. Eligible studies were RCTs in adults reporting gut microbiota outcomes following berberine supplementation. Methodological quality was assessed using the Cochrane Risk of Bias tool. Microbiota assessment methods, including sequencing platforms and bioinformatic pipelines, were systematically characterised across the included studies. **Results**: Seven RCTs enrolling 34 to 446 participants per intervention arm were included across diverse clinical populations—type two diabetes mellitus (T2DM), hyperlipidaemia, colorectal adenoma, psychiatric disorders, and Parkinson’s disease. Six of seven studies reported significant compositional shifts; the most extensively characterised changes—observed predominantly in T2DM populations—included enrichment of γ-Proteobacteria and depletion of butyrate-producing taxa, with specific taxa and the breadth of compositional changes varying considerably across clinical populations and sequencing methodologies. These shifts co-occurred with improvements in fasting glucose, lipid profiles, and inflammatory markers; however, causal inference cannot be established. **Conclusions**: Berberine consistently modulated gut microbial composition across heterogeneous clinical populations, with concurrent cardiometabolic and anti-inflammatory improvements. Compositional shifts were not uniformly favourable, and findings should be interpreted as hypothesis-generating. Geographically diverse, mechanistically focused RCTs are required to establish causality.

## 1. Introduction

Over the last two decades, remarkable advancements in gut microbiota research have increased our understanding of many diseases, bringing novel insights into various therapeutic approaches [1,2]. Dysbiosis, a disruption in the balance of the gut microbial community characterised by changes in microbial composition, metabolic processes, and/or intestinal localisation, disrupts normal gut function and homeostasis, with reduced diversity, overgrowth of harmful bacteria, or loss of beneficial taxa as its primary indicators [3].

The gut microbiota is extensively shaped by external factors, particularly dietary patterns [4], and individual responses to dietary interventions are strongly linked to shifts in microbial composition, underscoring the need for personalised nutritional approaches [5]. Alongside diet, inappropriate antibiotic use is associated with dysbiosis and chronic illnesses such as obesity, diabetes, and cardiovascular and renal diseases [6], while specific diseases, including colorectal cancer, are characterised by overgrowth of pathogenic bacteria, and others, like inflammatory bowel disease, by a marked reduction in health-associated populations [7].

These links have prompted exploration of nutritional interventions capable of restoring microbiota homeostasis [4], with evidence suggesting that the Mediterranean diet may confer protection against chronic disease through increased intake of prebiotics, (poly)phenols, omega-3 fatty acids, and probiotics [8].

The definition of prebiotics has expanded beyond non-digestible carbohydrates to encompass a broader range of bioactive compounds [9], including (poly)phenols, alkaloids [10,11], glucosinolates [11], and saponins [12], all investigated for their potential prebiotic effects [13,14].

Berberine, an isoquinoline alkaloid concentrated in the genus *Berberis* and widely used in traditional Chinese medicine, has attracted considerable attention for its pharmacological properties in cancer, inflammation, and metabolic syndrome [15,16]. Its association with the gut microbiota is an active research area [17]. Preclinical studies demonstrate enrichment of short-chain fatty acid (SCFA)-producing bacteria with anti-inflammatory properties [18], while clinical data indicate alterations in microbial composition alongside improvements in glucose and lipid metabolism [19,20]. As a compound bridging traditional medicine and contemporary clinical practice, berberine represents a compelling candidate for evidence-based integrative nutritional strategies targeting the gut microbiota. While berberine is the primary bioactive alkaloid of interest, its pharmacological activity in vivo is further modulated by gut microbiota-mediated biotransformation into active metabolites—including dihydroberberine and berberrubine—which exhibit distinct bioavailability profiles and may independently influence microbial composition and metabolic outcomes [21,22].

Beyond its microbiota-modulatory properties, berberine exerts direct cardiometabolic effects through multiple mechanisms. It activates AMP-activated protein kinase (AMPK), a master regulator of cellular energy homeostasis, thereby reducing hepatic glucose output, inhibiting gluconeogenesis, and improving insulin sensitivity [23,24]. Berberine also upregulates low-density lipoprotein receptor expression and inhibits proprotein convertase subtilisin/kexin type 9, contributing to reductions in low-density lipoprotein cholesterol (LDL-C) and total cholesterol [25]. Additionally, berberine attenuates systemic inflammation by suppressing nuclear factor kappa B pathway (NF-κB) signalling and reducing pro-inflammatory cytokines including tumour necrosis factor-alpha (TNF-α), interleukin (IL)-6, and C-reactive protein in a dose-dependent manner [26]. These direct pharmacological effects, acting in parallel and potentially synergistically with microbiota-mediated pathways, underpin the cardiometabolic outcomes observed in clinical trials and contextualise the integrative scope of the present review.

Among the microbiota-derived metabolites implicated in berberine’s systemic effects, SCFAs—particularly butyrate, propionate, and acetate—play a central mechanistic role. In this context, berberine has been shown to promote SCFA production through selective enrichment of SCFA-producing bacteria, including *Blautia* spp., with *Blautia*-mediated demethylation of berberine further stimulating butyrate and acetate synthesis via acetogenesis and cross-feeding [21,27]. These microbially derived metabolites contribute to intestinal barrier integrity, attenuation of lipopolysaccharide (LPS)-mediated inflammatory signalling, and improvement in insulin sensitivity [27], providing a plausible microbiota-dependent mechanism linking berberine supplementation to the cardiometabolic and anti-inflammatory outcomes observed in clinical trials.

Despite this growing interest in berberine research, a comprehensive systematic review synthesising evidence from randomised controlled trials (RCTs) remains lacking, limiting the understanding of the consistency, efficacy, and mechanisms through which berberine modulates gut microbial composition and function. Therefore, this systematic review aimed to evaluate the impact of berberine supplementation on gut microbiota in RCTs, with secondary objectives encompassing changes in cardiometabolic risk factors, inflammatory markers, immunological parameters, and neurodegenerative status.

Unlike previous reviews that have included preclinical data, observational studies, or mixed-design trials, the present review restricts its synthesis exclusively to randomised controlled trials in humans, thereby providing the first evidence-based appraisal of berberine’s microbiota-modulatory effects under controlled experimental conditions. Furthermore, this review introduces a systematic characterisation of the microbiota assessment methodologies employed across the included trials—including sequencing platforms, bioinformatic pipelines, and alpha diversity indices—and critically examines dose-dependent patterns of microbiota modulation, aspects not previously addressed in the berberine literature.

## 2. Materials and Methods

The protocol of this systematic review was registered in PROSPERO (registration number CRD42024524143). This systematic review was conducted and reported in accordance with the Preferred Reporting Items for Systematic Reviews and Meta-Analyses (PRISMA) guidelines [28]. The systematic review search was performed independently by two authors, while possible disagreements were handled by consensus.

### 2.1. Literature Search

The online literature search was conducted up to 10 January 2026 using four databases: PubMed, Web of Science, Scopus, and Embase. English language studies were included using the following key search query: (((berberine) AND (microbiota OR microbiome OR flora OR gut OR intestin*)) AND (randomise OR trial OR rct)) NOT (review).

### 2.2. Eligibility Criteria

The PICOS model (Population, Intervention, Comparison, Outcome, and Study design) was used to specify the eligibility criteria (Table 1). The inclusion criteria for the review were: (1) original studies published in English (upon screening, no records in other languages met the remaining eligibility criteria); (2) interventions that included supplementation with berberine (alone or in combination with supplements or medication); (3) randomised controlled clinical trial with either a crossover or a parallel trial design, lasting at least 2 weeks; (4) studies conducted in adult (age ≥ 18 years), non-pregnant participants (healthy or otherwise); and (5) studies that reported data on at least one of the designated outcome markers related to gut microbiota modulation. Exclusion criteria included: (1) studies with a duration < 2 weeks; (2) studies without randomisation or control or with insufficient information regarding the evaluated biomarkers; (3) studies with a lack of a proper control group that isolates the effects of berberine; (4) studies conducted in <18 years old or pregnant subjects.

### 2.3. Types of Outcomes

Primary outcomes of this systematic review encompassed the assessment of berberine’s impact on the gut microbiota, focusing on variations in the abundance of bacterial species, as well as relevant gut microbiota-related biomarkers, such as the Shannon diversity index. Secondary outcomes of this review included the evaluation of clinical parameters relevant to health status and cardiometabolic risk factors, such as triglycerides (TG), total cholesterol (TC), high-density lipoprotein cholesterol (HDL-C), LDL-C, fasting blood glucose, and homeostatic model assessment of insulin resistance (HOMA-IR), among others.

### 2.4. Data Extraction

The included studies were reviewed, and the following data were extracted: (1) publication details—name of the first author, year of publication and title, and country of publication; (2) study characteristics: study design, arm number, intervention duration, number of participants exposed to intervention, number of participants included in control groups, and dosage of the extract; (3) sample characteristics: country, number of males and females, health status, and medications; (4) information on reported outcomes.

### 2.5. Quality Assessment

Two reviewers independently assessed the methodological quality of the eligible studies using version 1 of the Cochrane Collaboration’s Risk of Bias tool (ROB1) [29]. This tool examines multiple domains, including: (1) random sequence generation (selection bias), (2) allocation concealment (selection bias), (3) the blinding of participants and personnel (performance bias), (4) the blinding of outcome assessment (detection bias), (5) incomplete outcome data (attrition bias), and (6) selective reporting (reporting bias). Each domain was rated as “yes,” “no,” or “unclear,” corresponding to low, high, and uncertain Risk of Bias, respectively.

## 3. Results

### 3.1. Study Selection

**Figure 1:**
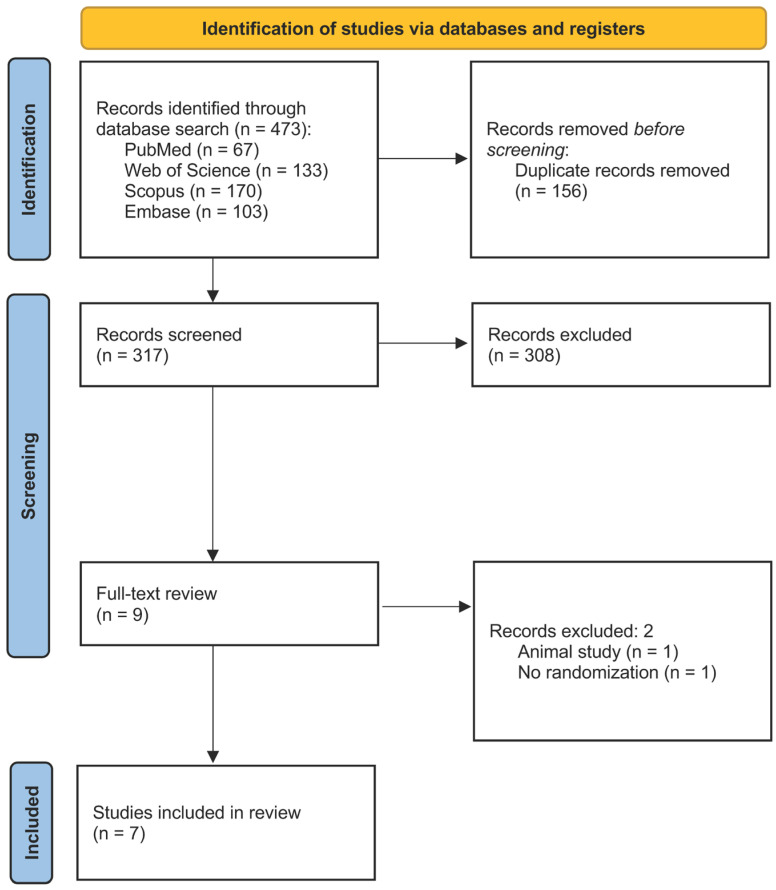
PRISMA flow diagram illustrating the study selection process.

Following the systematic database search and removal of duplicates, 317 records were screened, of which seven RCTs met the eligibility criteria and were included in this review (Figure 1). Two studies were excluded at the full-text stage: one conducted in animals [30] and one lacking a described randomisation method [31]. Finally, seven papers were found appropriate for inclusion and were analysed in this systematic review.

### 3.2. Article Characteristics

The seven included RCTs were all conducted in China, enrolling adult populations with varying clinical profiles: one study in colorectal adenoma [32], one in psychiatric and metabolic disorders [33], three in T2DM [34,35,36], one in hyperlipidaemia [37], and one in Parkinson’s disease [38] (Table 2). All trials used a randomised, placebo-controlled, parallel design; berberine hydrochloride was the predominant form used, with daily doses ranging from 0.1 g/day [33] to 1.2 g/day [34,35], and intervention durations from 12 to 104 weeks. Participant characteristics—including age, BMI, and sex distribution—were broadly comparable between intervention and control groups across trials. However, one study did not report the BMI average of the population included [38].

### 3.3. Microbiota Assessment Methods Across Included Studies

Substantial heterogeneity was identified in the microbiota assessment methods employed across the seven included trials (Table 3). Four studies utilised shotgun metagenomics [32,34,35,36], one study employed a dual-method approach combining both 16S rRNA amplicon sequencing and shotgun metagenomics [37], one study relied exclusively on 16S rRNA amplicon sequencing with OTU-based clustering against the SILVA database using the RDP Classifier [38], and one study assessed intestinal flora using targeted quantitative PCR (qPCR) limited to six predefined bacterial taxa [33]. Sequencing platforms varied across studies, including BGISEQ-500 [34,35], Illumina HiSeq [32], Illumina NovaSeq 6000 [36], and Illumina HiSeq 2500/X Ten [37], each differing in read length, throughput, and taxonomic resolution. Bioinformatic pipelines were similarly heterogeneous: shotgun metagenomic studies employed MetaPhlAn2 [36], SOAP2 [35,36,37], and BLASTX [34] for taxonomic profiling, while amplicon-based studies relied on QIIME with UniFrac distance matrices [37] and RDP Classifier [38]. Alpha diversity indices were reported inconsistently: the Shannon index was used in two studies [35,37]; the Ace, Chao, Shannon, and Simpson’s indices in one [38]; and alpha diversity was not assessed or reported in the remaining four studies [32,33,34,36].

### 3.4. Gut Microbiota Modulation After Berberine Treatment

The bacterial taxa discussed in the following section reflect those explicitly reported by the included studies and were not selected a priori by the review authors. No pre-specified taxonomic focus was applied; all taxa reported as significantly altered in the original trials are presented as described in the source studies.

The effects of berberine supplementation on alpha diversity were inconsistent across the included trials. A significant increase in alpha diversity was reported in patients with Parkinson’s disease following 12 weeks of supplementation at 0.6 g/day [38], whereas a significant decrease was observed in treatment-naïve T2DM patients receiving 1 g/day for 16 weeks [36]. Four studies found no significant effect on alpha diversity [32,34,35,37], though a reduction in gut microbial gene richness was noted in T2DM patients on antidiabetic therapy receiving 1.2 g/day for 12 weeks [35], and an increase in alpha diversity was observed specifically in hyperlipidaemic patients whose triglyceride levels decreased following supplementation [37]. Alpha diversity was not assessed in one study [33].

With the exception of one study [37], berberine supplementation significantly altered gut microbial composition compared with controls across all included trials, with the pattern of change varying by clinical population (Table 4) [32,33,34,35,36,37,38]. The most consistent and extensively characterised effects were observed in T2DM patients [34,35,36]. In this population, berberine at 1.2 g/day for 12 weeks modulated the relative abundance of 36 bacterial taxa compared with placebo, with a predominant reduction in butyrate-producing species—including *Faecalibacterium prausnitzii*, *Roseburia* spp., and *Bifidobacterium* spp.—and a concurrent enrichment of *Bacteroides* spp. and γ-Proteobacteria, including opportunistic pathogens such as *Klebsiella pneumoniae* and *Escherichia* spp. [35]. Consistent shifts were also observed within the berberine group from baseline to post-supplementation [35].

In a study using subjects from the same cohort, berberine alone significantly reduced *Bifidobacterium breve* abundance compared with berberine plus probiotics, and *Bifidobacterium longum* abundance compared with placebo [34]. Similarly, supplementation with 1 g/day for 16 weeks in treatment-naïve T2DM patients enriched Proteobacteria—including *Klebsiella pneumoniae*, *Escherichia* spp., *Ruminococcus torques*, *Ruminococcus gnavus*, and *Eubacterium ramulus*—while *Roseburia* spp. abundance declined [36].

In subjects with a history of colorectal adenoma, supplementation with 0.6 g/day of berberine for over one year significantly altered the abundance of multiple bacterial taxa compared with placebo, including reductions in *Veillonella parvula*, *Akkermansia muciniphila*, *Clostridium cellulovorans*, and *Eubacterium limosum* at the species level and *Anaerococcus*, *Clostridium*, *Solitalea*, *Pedobacter*, and *Roseburia* at the genus level [32]. In individuals with psychiatric disorders and antipsychotic-induced metabolic syndrome, 12 weeks of supplementation at 0.1–0.3 g/day reduced intestinal abundance of Firmicutes spp. and coliform bacteria, while *Bacteroides* spp. abundance also decreased [33]. In patients with Parkinson’s disease, supplementation with 0.6 g/day for 12 weeks produced significant shifts in beta diversity, with greater compositional changes being observed in the control group compared with the berberine group; however, specific taxa driving these differences were not reported [38].

### 3.5. Metabolic and Inflammatory Markers After Berberine Treatment

As pre-specified in the review protocol, cardiometabolic and inflammatory outcomes were designated as secondary endpoints, reported where available to contextualise the microbiota findings rather than to provide a standalone clinical efficacy synthesis. Accordingly, the following section summarises these outcomes descriptively, with critical appraisal limited to their co-occurrence with microbiota shifts.

Six of the seven included studies reported significant improvements in at least one biological parameter following berberine supplementation [33,34,35,36,37,38], with three identifying a direct correlation between these metabolic improvements and the microbiota-modulatory effects of berberine [34,35,37]. Regarding glucose homeostasis, berberine supplementation was associated with reductions in fasting plasma glucose [33,35,36], 2 h post-load plasma glucose [35,36], fasting plasma insulin [33], HOMA-IR [33], and HOMA-β [35]. In terms of lipid metabolism, significant reductions were observed in total cholesterol [35,36,37] and triglycerides [33,35,37], with additional decreases in LDL-C reported in two studies [35,37], HDL-C showed divergent responses, decreasing in one study [35] and increasing in another [36], while postprandial triglyceride levels were reduced in one study [34]. Inflammatory markers—including IL-6, IL-8, and TNF-α—were significantly reduced in the berberine group compared with placebo [38], alongside reductions in BMI and waist circumference [33]. Additional functional changes included alterations in bile acid metabolism [35], enhancement of carbohydrate metabolic pathways—including galactose, fructose, mannose, and glycerolipid metabolism [36]—and enrichment of xenobiotic biodegradation and metabolism pathways [36].

Notably, improvements in cardiometabolic markers were not uniformly observed across all populations, and the magnitude of effect varied considerably by clinical context, dose, and concomitant therapy. The heterogeneity in reported outcomes—spanning glycaemic markers, lipid profiles, and inflammatory mediators—reflects the diversity of the clinical populations included and limits cross-study comparisons of cardiometabolic efficacy.

### 3.6. Risk of Bias (RoB) Assessment

The RoB assessment using the Cochrane Collaboration’s ROB1 tool revealed variations in methodological rigor among the included studies (Table 5). All studies demonstrated low risk for random sequence generation, indicating the proper implementation of a randomisation method. However, allocation concealment was unclear in six out of seven studies, with only Ming et al. (2021) achieving a low risk rating [36]. The blinding of participants and personnel showed inconsistencies, with Pu et al. (2021) [33] and Li et al. (2022) [38] receiving high risk ratings, suggesting a greater potential for performance bias. Similarly, outcome assessment blinding was high-risk in these two studies, further raising concerns about detection bias. Incomplete outcome data and selective reporting were mostly rated low-risk, though Li et al. (2022) [38] and Qian et al. (2023) [32] had unclear assessments in these domains, indicating possible reporting issues. Overall, Ming et al. (2021) [36] was the most methodologically robust study, receiving low risk ratings across all domains. In contrast, Pu et al. (2021) [33] and Li et al. (2022) [38] exhibited the highest Risk of Bias, particularly due to inadequate blinding and unclear reporting. The remaining studies had uncertain overall risk, primarily due to missing details on allocation concealment and the blinding of outcome assessment.

## 4. Discussion

This systematic review synthesised evidence from seven RCTs examining the effects of berberine supplementation on gut microbiota composition and associated cardiometabolic, inflammatory, and neurological outcomes. The clinical populations included reflect the breadth of conditions in which gut microbiota dysbiosis has been implicated (from metabolic disorders and colorectal cancer to neurodegenerative and psychiatric disease [39,40,41]) underscoring the therapeutic relevance of microbiota-targeted interventions across heterogeneous disease contexts. Dysbiosis-associated endotoxaemia has been linked to insulin resistance, obesity, and T2DM [42], all recognised risk factors for tumour progression [43], while alterations in the gut–brain axis are known to influence neuro-inflammation, neurotransmitter synthesis, and brain function in both neurodegenerative and psychiatric disorders [44]. Against this background, the present findings indicate that berberine consistently modulates gut microbial composition across all these clinical contexts, despite exerting only modest and inconsistent effects on alpha diversity. Notably, six of seven studies reported concurrent improvements in cardiometabolic and inflammatory markers alongside shifts in gut microbial composition. However, given that these changes were observational and no studies were designed to establish microbiota-mediated causality, these findings should be interpreted as hypothesis-generating rather than mechanistically conclusive. Importantly, the compositional changes observed were not uniformly consistent with a conventionally favourable microbiota profile—particularly in T2DM populations, where berberine enriched γ-Proteobacteria, including *Klebsiella pneumoniae*, while reducing butyrate-producing taxa such as *Faecalibacterium prausnitzii* and *Roseburia* spp. This apparent paradox—whereby microbiota shifts of uncertain functional benefit co-occur with measurable metabolic improvements—suggests that berberine’s cardiometabolic effects may be driven by mechanisms that are partially independent of compositional microbiota changes, including direct modulation of bile acid metabolism, intestinal glucose absorption, and host inflammatory pathways.

In subjects with a history of colorectal adenoma, long-term supplementation with 0.6 g/day of berberine did not alter alpha diversity but significantly reduced the abundance of *Veillonella parvula* [32]—a taxon implicated in tumour progression through activation of nucleotide oligomerization domain 2/cellular communication network factor 4 (NOD2/CCN4/NF-κB) [45] whose lipopolysaccharides have been shown to disrupt colonic epithelial barrier integrity and promote intestinal dysbiosis [46]. This finding is particularly relevant given preclinical evidence of berberine’s anti-tumour activity, including reduced tumour volume and angiogenesis in animal models [47], and clinical data demonstrating decreased radiation-induced lung injury in non-small-cell lung cancer patients [48] and a lower risk of colorectal adenoma recurrence following polypectomy [49]. Collectively, these observations are consistent with the hypothesis that berberine may exert part of its chemopreventive potential through selective modulation of pro-tumourigenic microbial taxa; however, causal inference cannot be drawn from the available evidence, and mechanistically designed RCTs are required to confirm this relationship.

**Figure 2:**
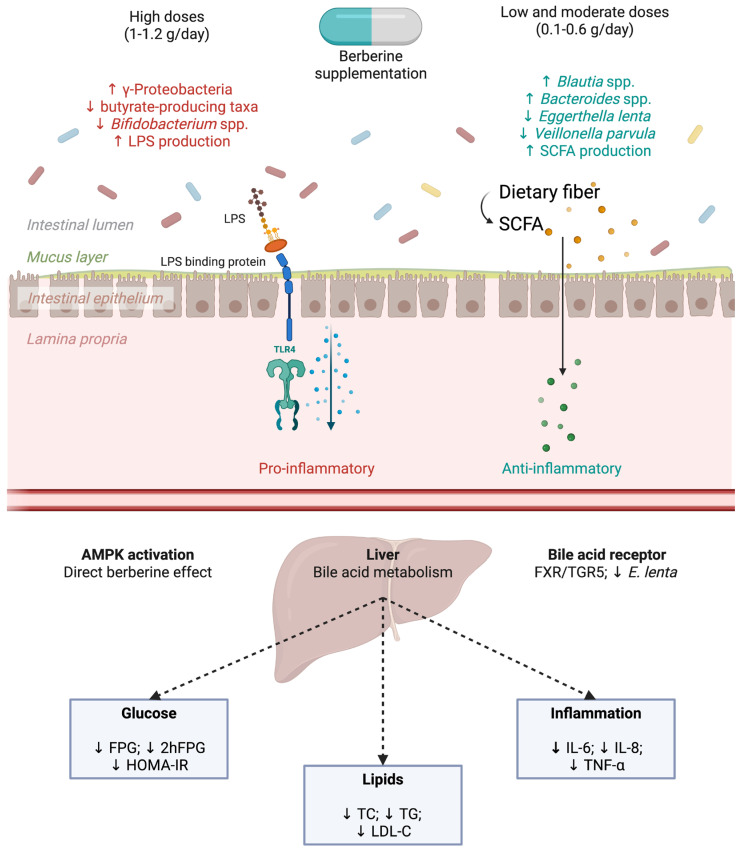
Proposed dose-dependent mechanisms underlying the cardiometabolic and gut microbiota-modulatory effects of berberine supplementation. Berberine may modulate gut microbial composition, LPS–TLR4 inflammatory signalling, SCFA production, bile acid metabolism, and systemic metabolic outcomes through both microbiota-dependent and direct pharmacological mechanisms. Higher doses may favour pro-inflammatory microbial shifts and increased LPS production, whereas low-to-moderate doses, particularly alongside dietary fibre, may support SCFA production and anti-inflammatory effects. Berberine may also act through AMPK activation and FXR/TGR5-related bile acid signalling, with downstream effects on glucose metabolism, lipid profile, and inflammatory markers. Co-occurrence of microbiota shifts and metabolic outcomes does not establish causality. Butyrate-producing taxa referenced in this figure include but are not limited to *Faecalibacterium prausnitzii*, *Roseburia* spp., and *Blautia* spp. SCFAs depicted include butyrate, acetate, and propionate, which collectively contribute to intestinal barrier integrity and attenuation of systemic inflammation. For the purpose of this figure, dose categories are defined based on the range of doses employed across the included trials: low dose: 0.1–0.3 g/day; moderate dose: 0.6 g/day; high dose: 1.0–1.2 g/day. These categories are intended to facilitate visual interpretation and do not represent established pharmacological thresholds. Arrows indicate proposed directional relationships between berberine supplementation, gut microbiota modulation, and downstream outcomes. Solid arrows represent direct pharmacological effects of berberine; dashed arrows represent proposed associations supported by clinical or preclinical evidence. All relationships depicted are hypothesis-generating and do not imply causality. Abbreviations: AMPK, adenosine monophosphate-activated protein kinase; FPG, fasting plasma glucose; 2hPG, 2 h post-load plasma glucose; FXR, farnesoid X receptor; HOMA-IR, homeostatic model assessment of insulin resistance; IL, interleukin; LDL-C, low-density lipoprotein cholesterol; LPS, lipopolysaccharide; SCFA, short-chain fatty acids; TC, total cholesterol; TG, triglycerides; TGR5, Takeda G protein-coupled receptor 5; TLR4, Toll-like receptor 4; TNF-α, tumour necrosis factor-alpha. Created in BioRender. Candrea, A. R. (2026); https://BioRender.com/j26rdff (accessed on 7 June 2026).

Among T2DM populations, berberine supplementation was associated with consistent cardiometabolic improvements alongside shifts in gut microbial composition, irrespective of concomitant antidiabetic therapy [34,35]. In patients receiving antidiabetic treatment, these concurrent changes included reduced microbial bile acid transformation, downregulation of related genes, and decreased *Eggerthella lenta* abundance—shifts that co-occurred with improvements in glycaemic and lipid markers [35]. The combination of berberine with *Bifidobacterium breve* was associated with lower postprandial lipid levels, suggesting a potentially synergistic interaction between berberine and probiotic supplementation at the microbiota–metabolite interface [34]. In treatment-naïve individuals, comparable metabolic improvements were observed alongside reduced microbial gene richness and shifts in the relative abundance of *Alistipes* and *Blautia* species [36,37], suggesting a functional compositional remodelling of the gut microbiome, though the mechanistic basis of these changes remains to be established. Notably, the T2DM-associated microbiota shifts also included enrichment of γ-Proteobacteria—including opportunistic pathogens such as *Klebsiella pneumoniae* and *Escherichia* spp.—alongside depletion of butyrate-producing taxa such as *Faecalibacterium prausnitzii* and *Roseburia* spp. and reductions in *Bifidobacterium longum* and *B. breve* [35,36]. This pattern, of uncertain functional significance, warrants careful interpretation. Importantly, not all compositional changes were unfavourable: concurrent enrichment of *Blautia* spp. [36], a genus associated with acetate production and cardiometabolic protection, suggests that berberine may simultaneously promote certain beneficial taxa and that the net functional impact of these complex compositional shifts cannot be reduced to a simple favourable/unfavourable dichotomy. Beyond its microbiota-modulatory effects, berberine has been shown to activate AMP-activated protein kinase (AMPK), a key regulator of cellular energy homeostasis, thereby directly reducing hepatic glucose output and improving insulin sensitivity through microbiota-independent pathways [27]. The microbiota-dependent mechanisms through which berberine may exert cardiometabolic benefits are equally relevant. By selectively modulating gut microbial composition, berberine influences the production of SCFAs—particularly butyrate and acetate—which reinforce intestinal barrier integrity, reduce LPS translocation into the portal circulation, and attenuate toll-like receptor 4 (TLR4)-mediated systemic inflammatory signalling [18]. Concurrently, berberine-associated reductions in *Eggerthella lenta* abundance contribute to decreased secondary bile acid transformation, with downstream effects on farnesoid X receptor (FXR) and Takeda G protein-coupled receptor 5 (TGR5) receptor signalling that may improve lipid and glucose homeostasis [23,35]. These microbiota-mediated and microbiota-independent pathways are not mutually exclusive and are likely to operate simultaneously, with their relative contributions varying across clinical populations, dosing regimens, and baseline microbiota composition. The proposed mechanisms underlying these findings are summarised in Figure 2.

It is further noted that all seven included trials administered berberine hydrochloride—a purified, standardised form of berberine isolated from plant sources—rather than crude botanical extracts, substantially minimising the confounding contribution of co-occurring isoquinoline alkaloids such as palmatine, coptisine, and jatrorrhizine, which are typically present in whole-plant preparations. Nevertheless, berberine’s pharmacological activity in vivo may be further modulated by gut microbiota-mediated biotransformation into active metabolites: the gut microbiota converts berberine into dihydroberberine via bacterial nitroreductases, with an intestinal absorption rate approximately five-fold greater than the parent compound [22], while berberrubine and thalifendine are produced through microbial demethylation—a process associated with stimulation of SCFA production via *Blautia* spp.-mediated acetogenesis [21]. These metabolites may independently influence microbial composition, bile acid metabolism, and glucose and lipid regulation, and their differential production across individuals with distinct microbiota profiles may partly account for the inter-study variability in berberine’s effects observed in the present review.

Moreover, in patients with antipsychotic-induced metabolic syndrome, berberine supplementation was associated with both metabolic improvements and a shift in microbial composition, including a reduction in *Firmicutes* and an increase in *Bacteroides* spp. [33], consistent with a potentially more favourable microbial profile in this population. However, the absence of formal correlation analyses between microbial and metabolic outcomes in this study precludes any mechanistic interpretation of these co-occurring changes.

The cardiometabolic findings of this review are consistent with those of a prior systematic review and meta-analysis demonstrating berberine’s efficacy in improving glycaemic parameters—including HbA1c, fasting plasma glucose, and HOMA-IR—and lipid profiles including TG, LDL-C, and HDL-C in T2DM [23]. Proposed mechanisms through which berberine may influence glucose and lipid metabolism via gut microbiota modulation include the enrichment of *Akkermansia*, *Eubacterium*, and *Ruminococcus* spp. in preclinical models [19], alongside reductions in faecal metabolites such as arbutin, isoleucine, and phenylalanine, and downregulation of the glycolysis/gluconeogenesis pathway [19]. Consistent with the bile acid-related findings observed in this review [35], berberine has also been described as a modulator of bile acid receptor expression and cholesterol-to-bile acid conversion, contributing to improved serum lipid levels [27]. Importantly, although an increase in Proteobacteria was observed in the clinical studies included in this review [35,36], preclinical evidence consistently reports the opposite pattern—a reduction in Proteobacteria abundance accompanied by decreased lipopolysaccharide production, improved intestinal permeability, and attenuation of metabolic endotoxaemia [27,50].

This discrepancy between preclinical and clinical findings highlights the complexity of translating animal model data to human populations and underscores the need for mechanistically focused RCTs. Experimental data further suggest a dose-dependent interaction between berberine and the gut microbiota, whereby higher doses may induce dysbiosis-like compositional changes while simultaneously improving intestinal integrity and inflammatory status [51], a pattern that may partly explain the paradoxical microbiota shifts observed in the included trials. This dose-dependent pattern is partially reflected in the clinical evidence synthesised in the present review. At the lowest doses employed (0.1–0.3 g/day), berberine produced only broad phylum-level shifts—reductions in Firmicutes and increases in Bacteroidetes—without identification of specific taxa, though this observation is also confounded by the use of targeted qPCR rather than comprehensive sequencing in that study [33]. At moderate doses (0.6 g/day), compositional changes were more selective and clinically relevant, including significant reductions in pro-tumourigenic *Veillonella parvula* following long-term supplementation [32] and improvements in alpha diversity alongside reductions in inflammatory markers in Parkinson’s disease patients [38]. At higher doses (1–1.2 g/day), the most extensive compositional remodelling was observed, encompassing modulation of up to 36 bacterial taxa [35], enrichment of γ-Proteobacteria and depletion of butyrate-producing taxa [35,36], and reductions in *Bifidobacterium* spp. [34,35]—a pattern consistent with the dysbiosis-like shifts described in preclinical high-dose models [51]. Additionally, variability in baseline microbial composition across participants may influence berberine bioavailability and pharmacological response [21], representing a significant source of inter-study heterogeneity. It should also be noted that in several included studies, berberine was administered alongside antidiabetic drugs or probiotics [34,35], which may independently influence gut microbiota composition and limit the attribution of observed effects to berberine alone. Collectively, these observations suggest a dose-dependent gradient in the breadth and directionality of berberine’s microbiota-modulatory effects, whereby lower doses may favour selective, potentially beneficial shifts, while higher doses induce broader compositional changes of uncertain functional significance. However, given the confounding effects of clinical population, sequencing methodology, and intervention duration, this dose-response relationship cannot be formally established from the available evidence and warrants systematic investigation in future mechanistically designed trials.

Moreover, the methodological heterogeneity in microbiota assessment across the included trials carries significant implications for the comparability and interpretation of findings. The contrast between hypothesis-free shotgun metagenomics [32,34,35,36,37] and targeted qPCR, which can only detect taxa for which primers have been designed a priori [33], means that the apparent absence of specific compositional shifts in one study [33] may reflect methodological limitation rather than genuine biological inactivity. Similarly, the use of different bioinformatic pipelines and reference databases across studies introduces additional uncertainty in taxonomic classification, particularly at the species level, where different tools may assign reads to different taxa. The sole study employing a dual-method approach, combining both 16S rRNA amplicon sequencing and shotgun metagenomics, provided the most comprehensive microbiota characterisation [37], highlighting that single-method approaches may underestimate the complexity of berberine-induced compositional shifts. Consequently, the apparent discordance in compositional findings across trials may be partly attributable to technical rather than biological variation, and these considerations highlight the urgent need for methodological standardisation in future RCTs, including unified sequencing platforms, consistent hypervariable region targeting, and harmonised bioinformatic pipelines, to enable meaningful cross-study comparisons of berberine’s effects on gut microbiota composition.

Beyond these methodological considerations, the clinical heterogeneity of the included populations also warrants individual discussion, particularly in conditions where the gut–brain axis plays a central mechanistic role, such as Parkinson’s disease [41]. In this context, the sole RCT identified in the present review suggests that berberine supplementation may improve alpha diversity and reduce inflammatory markers, including IL-6, IL-8, and TNF-α, in patients with Parkinson’s disease [38], consistent with a potential modulatory role along the gut–brain axis. These observations are further supported by a meta-analysis of RCTs demonstrating dose-dependent reductions in major inflammatory markers following berberine supplementation, with optimal effects at doses below 1000 mg/day over periods shorter than five weeks [26]. Preclinical evidence further implicates the gut–brain axis, with transplantation of *Enterococcus faecalis* or *Enterococcus faecium* shown to increase brain dopamine levels and alleviate Parkinsonian symptoms in mice—effects potentiated by berberine co-administration [52] Beyond dopamine metabolism, berberine has been shown to attenuate microbiota-related inflammatory pathways by reducing pro-inflammatory cytokines and trimethylamine-N-oxide levels while promoting SCFA-producing bacteria, including *Akkermansia muciniphila* [18]. These mechanistic insights, however, derive predominantly from preclinical models and require confirmation in well-designed clinical trials before their relevance to human Parkinson’s disease can be established.

## 5. Limitations

Several limitations of this review warrant consideration. Although only randomised studies were included, the lack of dietary and lifestyle control in several trials may have contributed to variability in outcomes; however, four of the seven studies incorporated lifestyle interventions as part of their design [34,35,36,37]. Substantial heterogeneity across included studies—in berberine dose (0.1–1.2 g/day), intervention duration (12–104 weeks), clinical populations, and reported outcomes—limits cross-study comparability and precluded the quantitative pooling of most outcomes. A further methodological source of heterogeneity relates to the microbiota assessment methods employed: studies utilising 16S rRNA amplicon sequencing and those employing shotgun metagenomics differ substantially in taxonomic resolution and sensitivity, which may account for some of the discordance in taxa reported across trials. Gut microbiota diversity and gene richness were not assessed in one study [33], and specific taxa altered by the intervention were not reported in another [38]. The use of the Cochrane ROB1 tool, rather than the updated ROB2, reflects the parallel-group design of all included trials and the suitability of ROB1 for this study type; however, it is acknowledged that ROB2 provides a more granular assessment of bias, and its application may have yielded different domain-level ratings for some studies. Moreover, all included studies were conducted in China, which substantially limits the generalisability of findings to other populations, given well-established regional and ethnic variation in gut microbiota composition [53,54]. Although some included studies reported participant sex distribution, sex-stratified analyses of gut microbiota outcomes remain limited across the included trials. The absence of sex-disaggregated microbiota data represents a meaningful gap, as potential differences in microbiota response to berberine supplementation between male and female participants cannot be adequately assessed from the available evidence. Finally, the relatively small number of eligible RCTs reflects the early stage of this research area and underscores the need for geographically diverse, methodologically standardised trials to consolidate the evidence base for berberine as a microbiota-targeted nutritional intervention.

## 6. Clinical Implications

The findings of this review carry several practical implications for clinicians and nutritionists integrating berberine into evidence-based therapeutic strategies. Among the clinical populations examined, the strongest and most consistent evidence supports the use of berberine as a microbiota-targeted adjunct intervention in T2DM, where supplementation at 1–1.2 g/day was associated with meaningful shifts in gut microbial composition alongside improvements in glycaemic and lipid markers, irrespective of concomitant antidiabetic therapy [34,35,36]. In hyperlipidaemic patients, berberine at 1 g/day appeared to confer microbiota-mediated lipid-lowering benefits particularly in individuals with elevated baseline triglycerides [37], suggesting that baseline metabolic profile may inform patient selection for berberine supplementation. In the context of colorectal adenoma prevention, long-term supplementation at 0.6 g/day showed selective modulation of pro-tumourigenic taxa, most notably *Veillonella parvula* [32], positioning berberine as a potentially valuable adjunct in post-polypectomy integrative care, though confirmatory RCTs are required before clinical recommendations can be made. In patients with antipsychotic-induced metabolic syndrome, lower doses (0.1–0.3 g/day) produced both metabolic and microbial improvements [33], suggesting tolerability at reduced doses in complex psychiatric settings. Finally, preliminary evidence supports berberine’s potential role in Parkinson’s disease management through gut–brain axis modulation, with improvements in alpha diversity and reductions in systemic inflammatory markers observed at 0.6 g/day [38].

Beyond microbiota modulation, the cardiometabolic benefits observed across the included trials underscore berberine’s genuinely integrative therapeutic profile. Reductions in fasting plasma glucose, 2 h post-load plasma glucose, and HOMA-IR were documented across T2DM and psychiatric populations [33,34,35], while significant improvements in total cholesterol, triglycerides, and LDL-C were reported in three trials [34,35,37]. Reductions in BMI and waist circumference were observed in the psychiatric population and anti-inflammatory benefits—evidenced by reductions in IL-6, IL-8, and TNF-α—were documented in Parkinson’s disease patients [38]. These findings collectively position berberine as an intervention whose clinical value spans glycaemic regulation, lipid management, and systemic inflammation—well beyond its microbiota-modulatory effects. This breadth of action, however, must be considered alongside an important clinical caveat: the enrichment of γ-Proteobacteria and depletion of butyrate-producing taxa observed at higher doses [35,36] represent a compositional shift of uncertain functional significance that warrants careful monitoring. Patients receiving berberine at doses ≥1 g/day—particularly those with pre-existing dysbiosis or compromised intestinal barrier function—should be assessed for potential adverse microbiota changes, and the benefit–risk balance should be evaluated on an individual basis [35,36].

## 7. Conclusions

This systematic review demonstrates that berberine consistently modulates gut microbial composition across heterogeneous clinical populations, with concurrent cardiometabolic and anti-inflammatory improvements observed in six of seven included trials. Compositional shifts were not uniformly favourable—particularly the enrichment of γ-Proteobacteria and depletion of butyrate-producing taxa in T2DM populations—and the co-occurrence of microbiota changes and metabolic outcomes should be interpreted as hypothesis-generating rather than mechanistically conclusive. The systematic characterisation of microbiota assessment methodologies across the included trials reveals substantial heterogeneity in sequencing platforms and bioinformatic pipelines, which substantially limits cross-study comparability and underscores the need for methodological standardisation. Collectively, berberine represents a clinically relevant, microbiome-targeted integrative intervention with population-specific therapeutic potential. The apparent paradox between unfavourable microbiota shifts and measurable cardiometabolic benefits identified in this review is, in itself, a research agenda—one that calls for trials designed not merely to replicate existing findings but also to interrogate the mechanistic hierarchy of microbial composition, metabolite production, and host metabolic response. To this end, the field now requires studies that stratify participants by baseline microbiota composition, standardise sequencing methodology, and incorporate mechanistic endpoints across diverse populations and dosing regimens—transforming berberine from a promising integrative compound into an evidence-based, precision nutritional intervention.

## Figures and Tables

**Table 1:** The PICOS model of eligibility criteria.

Population	Human Age ≥ 18 Non-pregnant Healthy or otherwise
Intervention	Berberine supplementation alone or in combination with other supplements or medication
Comparison	Placebo or other supplements medication alone
Outcome	Gut microbiota diversity (Simpson’s index/Shannon index/Chao index/Ace index) Abundance changes at the phylum/class/order/family/genus/species level Other metabolic changes, such as blood glucose, HbA1c, TG, HDL-C, LDL-C, TC, insulin levels, etc., if applicable
Study design	Randomised controlled clinical trials with either a crossover or a parallel design, lasting at least ≥2 weeks

HbA1c: glycated haemoglobin; TG: triglycerides; HDL-C: high-density lipoprotein cholesterol; LDL-C: low-density lipoprotein cholesterol; TC: total cholesterol.

**Table 2:** Characteristics of selected RCTs examining the effects of berberine supplementation on gut microbiota.

Lead Author, Year, Country	Study Design	Participants	Intervention	Duration of Study	Subjects’ Characteristics: Number of Participants (Intervention/Placebo), Sex (Males/Females), Age (Intervention/Placebo—Median or Mean), BMI (Intervention/Placebo—Median or Mean), Medication
Zhang et al., 2020, China [35]	Randomised, double-blinded, placebo-controlled, parallel trial	T2DM subjects	1.2 g	12 weeks	98/103 120/81 53/54 25.7/26.2 Oral antidiabetic agents, GLP-1 agonists, or insulin
Wu et al., 2022, China [37]	Randomised, double-blinded, placebo-controlled, parallel trial	Hyperlipidaemic subjects	1 g	12 weeks	42/41 37/46 51.89/56.14 26.07/25.56 No medication
Pu et al., 2021, China [33]	Randomised, placebo-controlled, parallel trial	Schizophrenia, bipolar disorder or schizoaffective psychosis and metabolic disorder subjects	0.1 to 0.3 g	12 weeks	58/52 79/31 44.31/43.34 24.68/24.44 Olanzapine
Ming et al., 2021, China [36]	Randomised, double-blinded, placebo-controlled, parallel trial	T2DM subjects	1 g	16 weeks	49/99 77/71 53.28/52.73 25.05/25.02 No medication
Li et al., 2022, China [38]	Randomised, placebo-controlled, parallel trial	Parkinson’s disease subjects	0.6 g	12 weeks	34/34 42/26 52.67/53.53 - Conventional treatment, anticholinergic drugs, amantadine, and dopamine receptor agonists
Wang et al., 2022, China [34]	Randomised, double-blinded, placebo-controlled, parallel trial	T2DM subjects	1.2 g	12 weeks	84/91 104/71 52.07/52.56 25.78/26.26 Oral antidiabetic agents, GLP-1 agonists, or insulin
Qian et al., 2023, China [32]	Randomised, placebo-controlled, parallel trial	Colorectal adenomas history subjects	0.6 g	2 years	429/462 580/311 57.4/56.6 24.1/24.2 No medication

BMI, body mass index; GLP-1, glucagon-like peptide-1; T2DM, type 2 diabetes mellitus.

**Table 3:** Microbiota assessment methods employed across included randomised controlled trials.

Study	Sequencing Type	Platform	Mode	Hypervariable Region	Taxonomic Pipeline
Qian et al. 2023 [32]	Shotgun metagenomics	Illumina HiSeq	Dual-end	N/A	FastQC, Trimmomatic, ea-utils; qPCR validation
Wang et al. 2022 [34]	Shotgun metagenomics	BGISEQ-500	100 bp paired-end	N/A	BLASTX vs. KEGG v76, SOAP denovo v1.05, reporter Z-scores
Zhang et al. 2020 [35]	Shotgun metagenomics	BGISEQ-500	100 bp paired-end	N/A	SOAP2.22 vs. IGC, KEGG KO, reporter Z-scores (≥1.96)
Ming et al. 2021 [36]	Shotgun metagenomics	Illumina NovaSeq 6000	151 bp paired-end	N/A	MetaPhlAn2, SOAP2 v2.22, KEGG annotation, reporter Z-scores
Wu et al. 2022 [37]	16S rRNA + shotgun metagenomics	Illumina HiSeq 2500/HiSeq X Ten	2 × 250 bp/2 × 150 bp	V3–V4 (16S only)	UCLUST, SILVA v128, QIIME, UniFrac; SOAP2, BLASTP vs. NR + KEGG
Li et al. 2022 [38]	16S rRNA amplicon sequencing *	Not specified	OTU-based	Not specified	RDP Classifier, Silva DB ≥ 80%, OTU clustering
Pu et al. 2021 [33]	Targeted qPCR	Real-time PCR	Predefined taxa only	N/A	N/A

* Sequencing method not explicitly stated by the authors; inferred from the use of OTU-based clustering, RDP Classifier, and Silva database, which are characteristic of 16S rRNA amplicon sequencing pipelines. N/A: not applicable; OTU: operational taxonomic unit; PCR: polymerase chain reaction; qPCR: quantitative polymerase chain reaction; 16S rRNA: 16S ribosomal ribonucleic acid; KEGG: Kyoto Encyclopedia of Genes and Genomes; KO: KEGG Orthology; IGC: integrated gene catalogue; SOAP: short oligonucleotide analysis package; FLASH: Fast Length Adjustment of Short Reads; UCLUST: USEARCH clustering algorithm; QIIME: Quantitative Insights Into Microbial Ecology; UniFrac: unique fraction metric; LEfSe: linear discriminant analysis effect size; LDA: linear discriminant analysis; PCoA: principal coordinates analysis; PLS-DA: partial least squares discriminant analysis; BLASTP: Basic Local Alignment Search Tool (protein); NR: NCBI non-redundant protein database; SILVA: ribosomal RNA sequence database; RDP: Ribosomal Database Project; V3–V4: hypervariable regions 3 and 4 of the 16S rRNA gene; bp: base pairs; PE: paired-end.

**Table 4:** Gut microbiota changes and secondary outcomes in included studies.

Lead Author, Year, Country	Gut Microbiota Changes	Secondary Outcomes
Zhang et al., 2020, China [35]	↑Shannon index and number of genes after antibiotic treatment BBR vs. placebo group: ↑*E. coli*, ↑*C. koseri*, ↑unclassified *Clostridium* sp. *HGF2*, ↑unclassified *Citrobacter* sp. *30 2*, ↑*K. variicola/pneumoniae*, ↑*E. aerogenes*, ↑*E. hormaechei/cloacae*, ↑*K. pneumoniae*, ↑*K. pneumoniae*/*Klebsiella variicola* group, ↑*R. gnavus*, ↑*E. cloacae*, ↑*K. oxytoca*, ↑*B. wadsworthia*, ↑*C. ramosum*, ↑*B. dorei*, ↑*B. dorei/vulgatus*, ↓*B. catenulatum Bpc*, ↓*C. perfringens*, ↓*B. adolescentis*, ↓*C. aerofaciens*, ↓*R. intestinalis*, ↓*R. hominis*, ↓*F. prausnitzii*, ↓*R. inulinivoans*, ↓*B. caccae*, ↓*B. longum*, ↓*C. bartletti*, ↓*E. lenta*, *↓E. eligens*, *↓R. bromi*, *↓V. parvula*, ↓*B. pectinophilus*, ↓*B. crossotus*, ↓unclassified *C.* sp. *L2-50*, ↓*C. eutactus*, ↓*P. merdae* Before and after in BBR: ↓*R. bromi*, ↓*A. shahii*, ↓*C. saccharolyticum*, ↓*O. sinus*, ↓*S. vestibularis*, ↓*P. capillosus*, ↓*S. thermophilus*, ↓*S. salivarius*, ↓*S. gordonii*, ↑*C. difficile*, ↑*B. dorei/vulgatus*, ↑*S. moorei*, ↑*B. clarus*, ↑*B. stercoris*, ↑*F. varium*, ↑Butyrate-producing bacteria	↓HbA1c ↓FPG ↓2hFPG ↓TG ↓TC ↓HDL-C ↓LDL-C ↑HOMA-ß
Wu et al., 2022, China [37]	↑Alpha diversity in subjects whose TG serum levels decreased	↓TG, ↓TC, ↓LDL-C
Pu et al., 2021, China [33]	BBR vs. placebo group: ↓*Firmicutes*, ↑*Bacteroides*, ↓*Coliform bacteria*. Before and after in BBR: ↓*Firmicutes*, ↑*Bacteroides*	↓FPG, ↓HbA1c, ↓TG, ↓BMI, ↓waist circumference, ↓FPI, ↓HOMA-IR
Ming et al., 2021, China [36]	Gene richness, before and after in BBR: ↓Before and after in BBR compared with placebo. Species level: ↑*K. pneumoniae*, ↑*E. unclassified*, ↑*R. torques*, ↑*R. gnavus*, ↑*E. ramulus*, ↓*R. inulinivorans*, ↓*R. intestinalis.* Genus level: ↑*Klebsiella*, ↑*Blautia*, *↑Lactobacillus*, ↑*Candidatus Saccharibacteria* (no name, unclassified), ↓*Roseburia.* Phylum level: ↑*Proteobacteria*, ↑*Candidatus Saccharibacteria*, ↓*Firmicutes*	↓2hFPG, ↓TC ↓HDL-C
Li et al., 2022, China [38]	BBR vs. placebo group: ↑Chao index, ↑Ace index, ↑Shannon index, ↓Simpson’s index	↓IL-8 ↓IL-6 ↓TNF-α
Wang et al., 2022, China [34]	↓*B. breve*, *↓B. longum*	↓TG
Qian et al., 2023, China [32]	No differences in alpha diversity (Shannon–Wiener index and Simpson’s index). Changes at the species level in BBR vs. placebo group: *↓V. parvula*, *A. muciniphila*, *C. cellulovorans*, *E. limosum.* Changes at the genus level in BBR: *Anaerococcus*, *Clostridium*, *Solitalea*, *Pedobacter*, *Roseburia*	Increased abundance of *V. parvula* might promote the progression of adenoma–carcinoma.

BBR, berberine; BMI, body mass index; FPG, fasting plasma glucose; FPI, fasting plasma insulin; HbA1c, glycated haemoglobin; HDL-C, high-density lipoprotein cholesterol; HOMA-β, homeostatic model assessment of beta-cell function; HOMA-IR, homeostatic model assessment of insulin resistance; IL-6, interleukin-6; IL-8, interleukin-8; LDL-C, low-density lipoprotein cholesterol; TC, total cholesterol; TG, triglycerides; TNF-α, tumour necrosis factor-alpha; 2hFPG, 2 h post-load plasma glucose; vs., versus. Note: ↑ indicates an increase; ↓ indicates a decrease. Bacterial taxa are reported as described in the original studies. Abbreviated species names follow standard binomial nomenclature after the genus has been introduced.

**Table 5:** Risk of Bias assessment according to the Cochrane Collaboration’s Risk of Bias assessment tool.

Study, Year (Reference)	Random Sequence Generation	Allocation Concealment	Blinding of Participants and Personnel	Blinding of Outcome Assessment	Incomplete Outcome Data	Selective Reporting	Overall Assessment of Risk of Bias
Zhang et al., 2020 [35]	Low	Unclear	Low	Unclear	Low	Low	Unclear
Ming et al., 2021 [36]	Low	Low	Low	Low	Low	Low	Low
Pu et al., 2021 [33]	Low	Unclear	High	High	Low	Low	High
Li et al., 2022 [38]	Low	Unclear	High	High	Unclear	Unclear	High
Wu et al., 2022 [37]	Low	Unclear	Low	Unclear	Low	Low	Unclear
Wang et al., 2022 [34]	Low	Unclear	Low	Unclear	Low	Low	Unclear
Qian et al., 2023 [32]	Low	Unclear	Low	Unclear	Unclear	Unclear	Unclear

## Data Availability

No new data were generated or analysed in this study. All data included in this systematic review were extracted from previously published articles and are presented within the manuscript.

## Abbreviations

The following abbreviations are used in this manuscript:

AMP	adenosine monophosphate
AMPK	AMP-activated protein kinase
BBR	berberine
BGISEQ-500	Beijing Genomics Institute sequencing platform 500
BLASTP	Basic Local Alignment Search Tool (protein)
BLASTX	Basic Local Alignment Search Tool (translated nucleotide)
BMI	body mass index
CCN4	cellular communication network factor 4
FBG	fasting blood glucose
FLASH	Fast Length Adjustment of Short Reads
FPI	fasting plasma insulin
FPG	fasting plasma glucose
FXR	farnesoid X receptor
GLP-1	glucagon-like peptide-1
HbA1c	glycated haemoglobin
HDL-C	high-density lipoprotein cholesterol
HOMA-β	homeostatic model assessment of beta-cell function
HOMA-IR	homeostatic model assessment of insulin resistance
IGC	integrated gene catalogue
IL-6	interleukin-6
IL-8	interleukin-8
KEGG	Kyoto Encyclopedia of Genes and Genomes
KO	KEGG Orthology
LDA	linear discriminant analysis
LDL-C	low-density lipoprotein cholesterol
LDLR	low-density lipoprotein receptor
LEfSe	linear discriminant analysis effect size
LPS	lipopolysaccharide
NCBI	National Center for Biotechnology Information
NF-κB	nuclear factor kappa B
NOD2	nucleotide oligomerization domain 2
NR	NCBI non-redundant protein database
OTU	operational taxonomic unit
PCR	polymerase chain reaction
PCoA	principal coordinates analysis
PCSK9	proprotein convertase subtilisin/kexin type 9
PICOS	Population, Intervention, Comparison, Outcome, Study design
PLS-DA	partial least squares discriminant analysis
PRISMA	Preferred Reporting Items for Systematic Reviews and Meta-Analyses
PROSPERO	International Prospective Register of Systematic Reviews
qPCR	quantitative polymerase chain reaction
QIIME	Quantitative Insights Into Microbial Ecology
RCT	randomised controlled trial
RDP	Ribosomal Database Project
RNA	ribonucleic acid
ROB	Risk of Bias tool
SCFA	short-chain fatty acid
SGLT1	sodium-glucose cotransporter 1
SILVA	Ribosomal RNA sequence database
SOAP2	Short Oligonucleotide Analysis Package version 2
T2DM	type 2 diabetes mellitus
TC	total cholesterol
TG	triglycerides
TGR5	Takeda G protein-coupled receptor 5
TLR4	Toll-like receptor 4
TMAO	trimethylamine N-oxide
TNF-α	tumour necrosis factor-alpha
UCLUST	USEARCH clustering algorithm
USEARCH	ultrafast sequence analysis tool
2hFPG	2 h post-load plasma glucose
16S rRNA	16S ribosomal ribonucleic acid
